# Transcriptomic analysis of wheat reveals possible resistance mechanism mediated by *Yr10* to stripe rust

**DOI:** 10.1007/s44154-023-00115-z

**Published:** 2023-10-23

**Authors:** Zhongyi Wu, Gaohua Zhang, Ran Zhao, Qi Gao, Jinchen Zhao, Xiaoxu Zhu, Fangyan Wang, Zhensheng Kang, Xiaojing Wang

**Affiliations:** 1 State Key Laboratory of Crop Stress Biology for Arid Areas and College of Life Sciences, Northwest A&F University, Yangling, 712100 Shaanxi China; 2 State Key Laboratory of Crop Stress Biology for Arid Areas and College of Plant Protection, Northwest A&F University, Yangling, 712100 Shaanxi China; 3 State Key Laboratory of Crop Stress Biology for Arid Areas and College of Natural Resources and Environment, Northwest A&F University, Yangling, 712100 Shaanxi China

**Keywords:** Transcriptome, *Yr10*, Virus-induced gene silencing system, (VIGS) system, *4-hydroxyphenylpyruvate dioxygenase*, HPPD

## Abstract

Stripe rust, caused by *Puccinia striiformis* f. sp. *tritici* (*Pst*), is a catastrophic disease that threatens global wheat yield. *Yr10* is a race-specific all-stage disease resistance gene in wheat. However, the resistance mechanism of *Yr10* is poorly characterized. Therefore, to elucidate the potential molecular mechanism mediated by *Yr10*, transcriptomic sequencing was performed at 0, 18, and 48 h post-inoculation (hpi) of compatible wheat Avocet S (AvS) and incompatible near-isogenic line (NIL) AvS + *Yr10* inoculated with *Pst* race CYR32. Respectively, 227, 208, and 4050 differentially expressed genes (DEGs) were identified at 0, 18, and 48 hpi between incompatible and compatible interaction. The response of *Yr10* to stripe rust involved various processes and activities, as indicated by the results of Gene Ontology (GO) enrichment analysis and Kyoto Encyclopedia of Genes and Genomes (KEGG) pathway analysis. Specifically, the response included photosynthesis, defense response to fungus, metabolic processes related to salicylic acid (SA) and jasmonic acid (JA), and activities related to reactive oxygen species (ROS). Ten candidate genes were selected for qRT-PCR verification and the results showed that the transcriptomic data was reliable. Through the functional analysis of candidate genes by the virus-induced gene silencing (VIGS) system, it was found that the gene *TaHPPD* (*4-hydroxyphenylpyruvate dioxygenase*) negatively regulated the resistance of wheat to stripe rust by affecting SA signaling, *pathogenesis-related* (*PR*) gene expression, and ROS clearance. Our study provides insight into *Yr10-*mediated resistance in wheat.

**Supplementary Information:**

The online version contains supplementary material available at 10.1007/s44154-023-00115-z.

## Introduction

Bread wheat (*Triticum aestivum* L.) is a major crop worldwide and is consumed as a staple food by 30% of the global population (FAOSTAT 2021). Stripe rust, caused by the fungal pathogen *Puccinia striiformis* f*.* sp*. tritici* (*Pst*), is a severe disease which is widespread in wheat-producing regions globally. Stripe rust generally results in yield losses of 10–70%, and up to 100% in severe cases (Chen et al. 2005; Chang et al. 2013). *Pst* is highly genetically diverse, resulting in the formation of different races with varying levels of pathogenicity (Wan et al. 2007). Increased virulence results in the weaking of existing plant resistance, with wheat varieties often losing their innate resistance to stripe rust after only 3–5 years of field use (Kang et al. 2015; Chen et al. 2013). The artificial breeding of varieties with long-lasting and broad-spectrum resistance is considered the most effective approach to disease control.

The plant innate immune system has evolved to effectively resist potentially harmful microorganisms, and there are two primary categories of resistance. The first is known as seedling or all-stage resistance (*R* gene-mediated resistance), which provides high-level resistance across all stages of plant growth. The second, adult plant resistance (APR), provides durable resistance to a variety of pathogens (Dinglasan et al. 2022). To date, more than 80 stripe rust resistance (*R*) genes have been classified in wheat (Gessese et al. 2019). *R* gene-encoded proteins recognize and interact with specific *Avr* gene-encoded pathogenic effector proteins to induce effector-triggered immunity (ETI) (Boller and He 2009). ETI is usually accompanied by a hypersensitive response (HR), which limits pathogenic spread by rapidly invoking programmed cell death at the infection site (Dangl et al. 2013).

In order to elucidate the molecular mechanism of wheat rust resistance, it is necessary to clone and identify *R* genes. Wheat resistance to stripe rust can be improved by aggregating multiple resistance genes (Bokore et al. 2017). In previous research, Avocet S was selected as a candidate recurrent parent to introduce a single gene, such as wheat rust resistance gene *Yr10*, utilizing traditional backcross methods in the establishment of a near-isogenic line (NIL). *Yr10* still showed resistance to certain races of *Pst*, such as CYR32 which is one of the prevalent races in China (McIntosh et al. 2018). Recently, the resistance conferred by *Yr10* were found to be overcome by the highly virulent *Pst* race CYR34 (V26) (McIntosh et al. 2018). In response, breeders have developed wheat varieties combining *Yr10* with other *R* genes in order to provide effective resistance to stripe rust.

Transcriptomic sequencing was introduced in 2004 (Bennett 2004), offering a highly sensitive and comprehensive approach to transcriptomic analysis. Advances in this field have led to the development of new methods and techniques, such as single-cell RNA sequencing (scRNA-seq) and meta-cluster analysis, that enable a deeper understanding of complex biological processes. The latest RNA sequencing and analysis technologies have been used to uncover the role of *CPR5* in plant immunity, as well as other potential candidate genes involved in the development of improved maize varieties (Peng et al. 2022; Xu et al. 2021). Pathogen infection leads to changes in gene and protein expression in host plants. In recent years, transcriptomic sequencing has been widely used to analyze changes in genes expression related to the wheat-pathogen interaction (Dobon et al. 2016; Yadav et al. 2016). For example, the mechanism of stripe rust resistance was revealed by studying transcriptional changes occurring in mature ‘Xingzi9104’ (XZ) wheat during *Pst* infection (Hao et al. 2016). The results suggested that APR mediates *Pst* resistance through several complex biological processes, such as reactive oxygen species (ROS) production, lignification, and sugar accumulation, among others (Hao et al. 2016). Specifically, mature plants were found to continually accumulate ROS in order to inhibit fungal growth and development. In another study, Zeng (2016) explored the *Yr26* gene mediated molecular interaction mechanism in *Pst*-inoculated wheat and identified a total of 14,449 genes with significant differential expression, many of which activated the salicylic acid (SA) pathway and inhibited the jasmonic acid (JA) pathway.

Previous studies on the *Yr10* gene have primarily focused on molecular markers, while a comprehensive characterization of the molecular mechanism underlying *Yr10*-mediated resistance, or of the pathways involving this gene, is lacking. In this study, RNA sequencing (RNA-seq) was used to analyze differential gene expression in Avocet S (AvS) and near-isogenic AvS + *Yr10* wheat. In our previous research, H_2_O_2_ had begun accumulated in guard cells and necrotic cell had appeared at the infection site at 18-24hpi in the incompatible interaction. And at 48hpi, the accumulation of H_2_O_2_ spread to neighboring cells and necrotic cell increased in the incompatible interaction. In contrast, no H_2_O_2_ generation could be detected and no necrosis occurred at this time in most of the infection sites in compatible interaction (Wang et al. 2007; Zhu et al. 2021). In conclusion, we considered that 0, 18, and 48 h are the key time points for wheat fighting against *Pst* and were selected as the three time points for RNA sequencing (RNA-seq) samples. Differentially expressed genes (DEGs) were identified, and their functions and associated pathways were characterized through Gene Ontology (GO) and Kyoto Encyclopedia of Genes and Genomes (KEGG) analyses. The function of the candidate gene *TaHPPD* (4-hydroxyphenylpyruvate dioxygenase) was verified through Virus-Induced Gene Silencing (VIGS), revealing that *TaHPPD* may be a negative regulator of stripe rust resistance in wheat. Specifically, *TaHPPD* may regulate the SA signaling pathway and *pathogen-related* (*PR*) genes expression. The results of this study will be foundational to future studies of the mechanism through which *TaHPPD* promotes stripe rust infection in wheat. In addition, these results will facilitate the precision breeding of wheat varieties with enhanced resistance to stripe rust.

## Results

### High-throughput sequencing of AvS and AvS + *Yr10 *infected with *Pst*

Three biological replicates of AvS and near-isogenic AvS + *Yr10* wheat were selected for transcriptomic sequencing at 0, 18, and 48 hpi with *Pst* race CYR32. A total of 624, 835, 643, raw reads were obtained from the sequencing of 18 leaf samples. After removing the linker-containing and low-quality sequences, 593, 577, 368, clean reads (89.04 Gb) were obtained. As shown in Table S1, the Q20 quality scores were ≥ 98.89%, and the GC content was 51.11–54.03%, indicating that the sequencing quality was good. The clean reads were assembled to the ‘Chinese Spring’ wheat reference genome (IWGSC RefSeq v1.0). The comparison rate ranged from 66.36% to 76.08%. Taken together, these results suggest that the selection of reference genome for transcriptomic sequencing was appropriate, resulting in a high quality alignment.

### DEGs between AvS and AvS + *Yr10 *infected with *Pst*

**Fig. 1:**
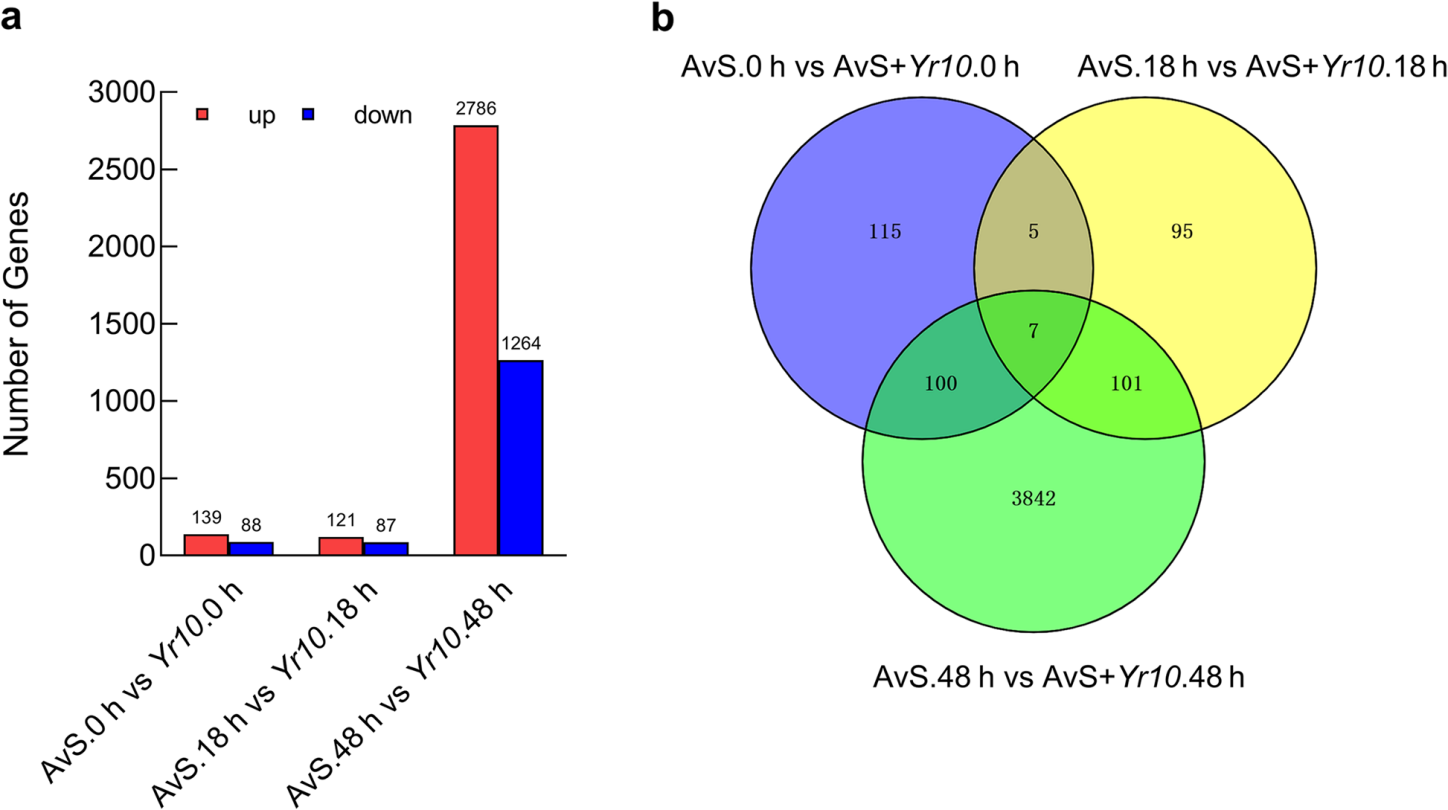
Statistical chart and Venn diagram of the DEGs at the three time points of DEGs after *Pst* infection between AvS vs AvS + *Yr10* samples. **a** Statistical chart of the DEGs. DEGs were identified by filtering the two-fold up-regulated and down-regulated genes with FDR ≤ 0.001. The rectangles represent the number of up-regulated (red) and down-regulated (blue) genes. **b** Venn diagram of the DEGs. These genes were obtained from the AvS.0 h vs AvS + *Yr10*.0 h, AvS.18 h vs AvS + *Yr10*.18 h, and AvS.48 h vs AvS + *Yr10*.48 h comparisons

A total of 227, 208, and 4050 DEGs ( RPKM > 1, |log_2_ Fold-Change|> 2, and FDR < 0.001) were obtained at 0, 18, and 48 hpi, respectively (Fig. 1a). Among them, there were 139, 121, and 2786 up-regulated genes and 88, 87, and 1264 down-regulated genes between compatible interaction (AvS/CYR32) and incompatible interaction (AvS + *Yr10*/CYR32) samples, respectively. We used cluster heat map to show the general expression pattern of DEGs among three time points (Fig. S1). The sharp increase in the number of DEGs observed at 48 hpi led us to speculate that this time point is critical for the expression plant defense-related genes in response to *Pst* infection.

In the comparison of AvS.0 h vs. AvS + *Yr10*.0 h and AvS.18 h vs. AvS + *Yr10.*18 h, 12 DEGs exhibited overlapping expression (Fig. 1b). Between AvS.0 h vs. AvS + *Yr10*.0 h and AvS.48 h vs. AvS + *Yr10*.48 h, 107 DEGs exhibited overlapping expression. Similarly, 108 overlapping DEGs were observed between AvS.18 h vs. AvS + *Yr10*.18 h and AvS.48 h vs. AvS + *Yr10*.48 h (Fig. 1b). Only 7 common DEGs were identified at all three time points (Fig. 1b).

### GO enrichment analysis of DEGs

The DAVID online website was utilized for GO enrichment analysis in order to identify key node genes related to plant disease resistance or susceptibility for subsequent functional analysis. The DEGs were found to be enriched in three categories of GO terms: molecular functions, biological processes, and cellular components (File S1).

**Fig. 2:**
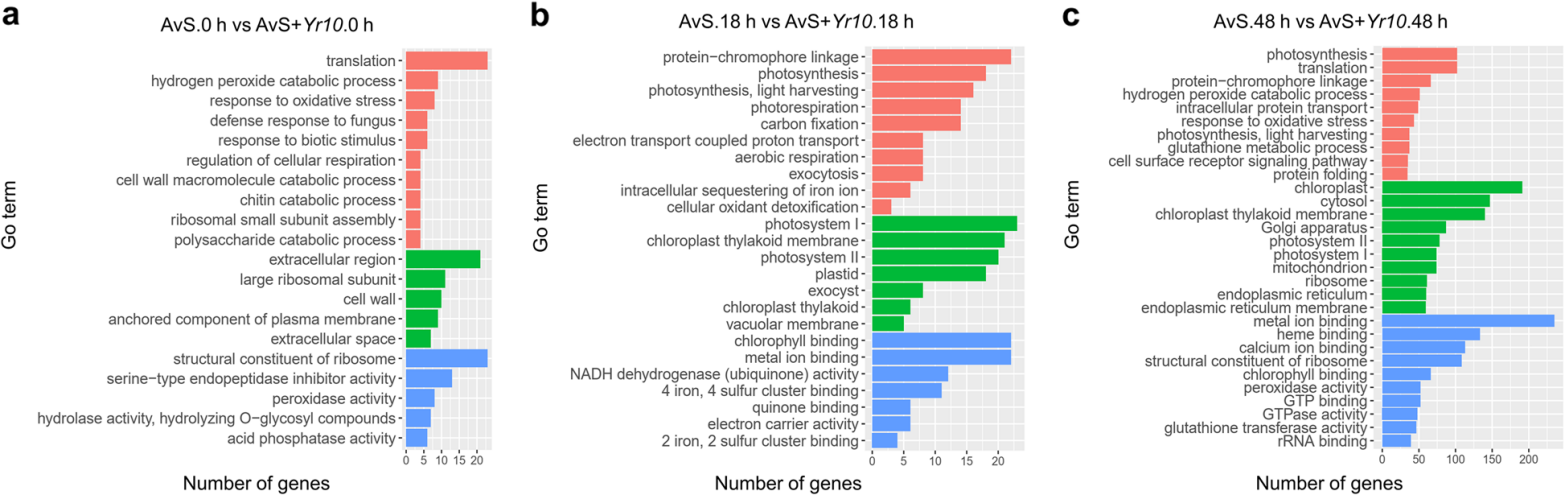
Statistical chart of GO terms during *Pst* CYR32 infection at three different time points between AvS and AvS + *Yr10* samples. **a**, **b**, and **c** show the statistical chart of GO terms during *Pst* CYR32 infection at three different time points (0 hpi, 18 hpi, 48 hpi) between AvS and AvS + *Yr10* wheat, respectively. The x-axis represents the number of DEGs enriched in this term. Red represents the biological processes, green represents cellular components and blue represents molecular functions

The results for AvS.0 h vs. AvS + *Yr10*.0 h and the AvS.18 h vs. AvS + *Yr10*.18 h can be found in File S1 and Fig. 2a and b. At 48 hpi, a total of 255 GO terms were enriched, far more than 27 and 36 GO terms at 0 hpi and 18 hpi, respectively. The GO terms enriched at 48 hpi included multiple aspects in Fig. 2c. In the “biological process”, terms such as response to oxidative stress (GO:0006979) and cell surface receptor signaling pathway (GO:0007166) were enriched, which may involve in plant immune response. In the “cellular component” category, diverse cell organelles like chloroplast (GO:0009507), Golgi apparatus (GO:0005794) and mitochondrion (GO:0005739) were enriched which indicates that the interaction between wheat and rust was so complicated that included multiple organelles to participate. In the “molecular function” category, various terms were enriched. Interestingly, terms like metal ion binding (GO:0046872) and calcium ion binding (GO:0005509) drew our attention as critical parts in plant signaling and metabolism. Further, peroxidase activity (GO:0004601) and chitinase activity (GO:0004568) which directly participate in plant immune were also enriched. Taken together, we recognize 48 hpi as the critical time points in our experiment. In both AvS.18 h vs. AvS + *Yr10*.18 h and AvS.48 h vs. AvS + *Yr10*.48 h, 9 significantly enriched GO terms (Q value ≤ 0.05) were found to be enriched in the biological process category and 5 terms were enriched in the molecular function category (Fig. S2). No terms were enriched across all three time points within either category (Fig. S2).

### KEGG enrichment analysis of DEGs

**Fig. 3:**
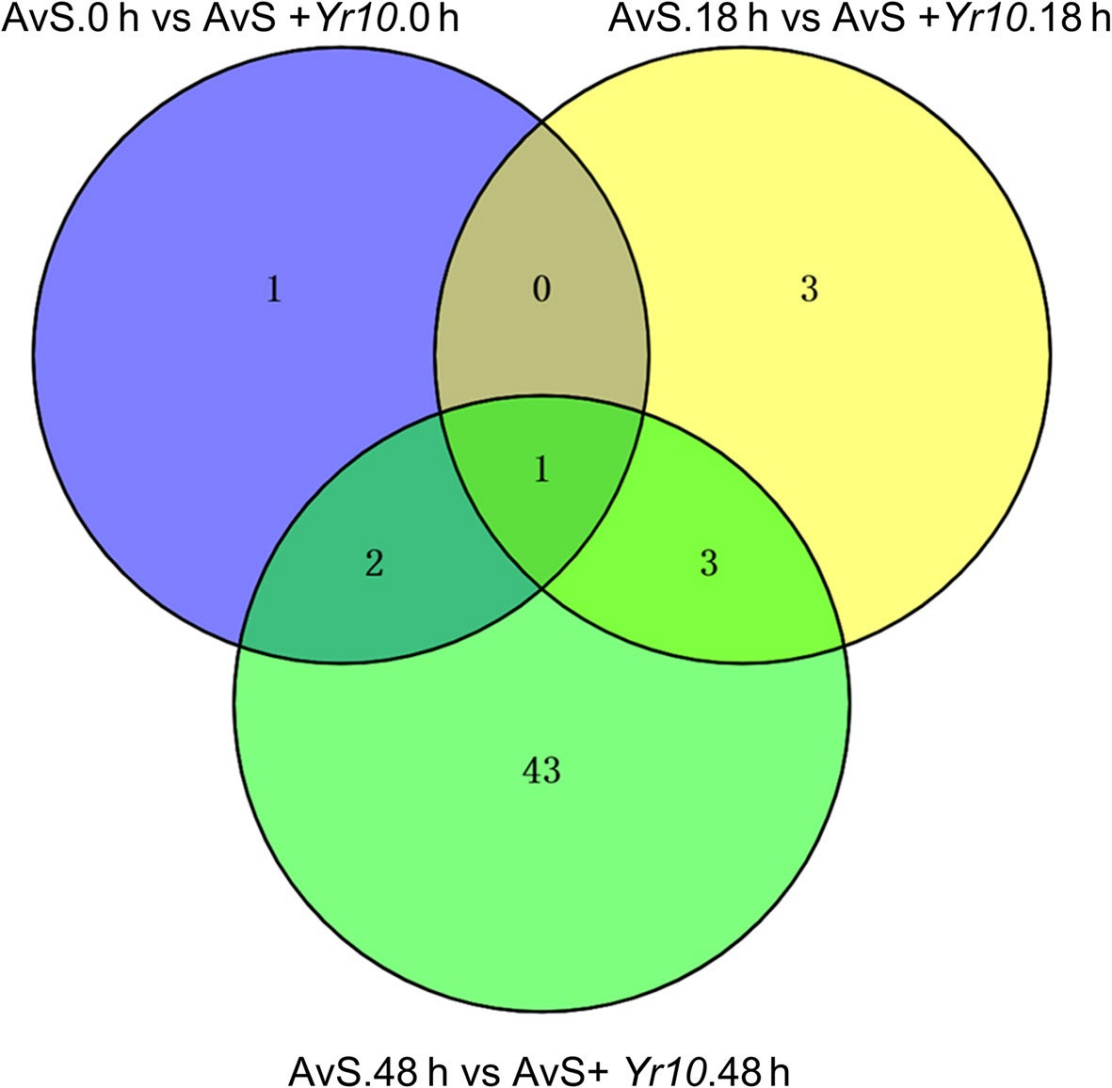
Venn diagram of the KEGG pathways. These pathways were obtained from the AvS.0 h vs AvS + *Yr10*.0 h, AvS.18 h vs AvS + *Yr10*.18 h, and AvS.48 h vs AvS + *Yr10*.48 h comparisons by filtering FDR-corrected Q-values ≤ 0.05 as the threshold

To further explore the metabolic pathways associated with the identified DEGs, all DEGs were annotated and subjected to KEGG analysis. The data indicated that there were 4, 7, and 49 pathways which were significantly (E-value < 0.05) enriched at 0, 18, and 48 hpi, respectively (File S1, Fig. 3, Fig. S3). The number of pathways that were significantly enriched at 48 hpi was greater than at 0 hpi and 18 hpi. Photosynthesis was enriched across all three time points. Nitrogen metabolism and ribosome were enriched at 0 hpi and 48 hpi. Glyoxylic acid and dicarboxylic acid metabolism, carbon fixation in photosynthetic organisms, and photosynthesis-antenna protein were enriched at 18 hpi and 48 hpi. As the 48hpi considered the most critical one among the three time points, we found that besides the pathways directly related to defense such as Melanogenesis and Plant-pathogen interaction, multiple metabolism pathways like Glycolysis/Gluconeogenesis and Fructose and mannose metabolism were also enriched. This indicated the sophisticated interaction network between rust and wheat included various aspects of wheat metabolism.

### Transcript levels of genes involved in the SA and JA defense pathways in the response to *Pst* infection

During the process of plant biotic stress response, the phytohormones SA and JA interact within a complex and cooperative interaction network. In general, both SA and JA interact in a mutually antagonistic fashion in response to pathogen attack (Lorenzo and Solano 2005; Mur et al. 2013). Here, we analyzed the transcript levels of genes involved in SA and JA signaling to study the *Yr10*-mediated defense network (Table 1). First, *phenylalanine ammonia-lyase* (*PAL*) genes, involved in SA synthesis, were up-regulated in AvS + *Yr10* at 0 hpi and 48 hpi. This expression pattern validates our previous finding that endogenous SA levels were higher during incompatible interactions than during compatible interactions (Zhu et al. 2021). Further, SA receptor NPR1 was slightly up-regulated, and SA-responsive PR proteins PR1, PR2, and PR5 also exhibited a similar expression pattern to *PAL*, indicating that SA and its downstream regulation network may be a major defense process in *Yr10-*induced immunity against *Pst*. Moreover, these genes were up-regulated at 0 hpi, suggesting that certain defense responses may have been pre-activated.

**Table 1:** Transcript levels of genes in wheat involved in SA and JA pathways

Category	Gene Description	Gene ID	Log_2_ Fold Change		
			0 h	18 h	48 h
SA	*NPR1*	TraesCS3B01G123800.1	-	0.801143	0.564811
		TraesCS3A01G298800.1	-	0.893744	0.984634
		TraesCS3D01G302900.1	0.144492	0.657611	0.835347
	*PR1*	TraesCS5B01G181500.1	3.062775	-0.46027	2.9987538
		TraesCS5A01G183300.1	2.482122	-0.35579	2.5565597
	*PR2*	TraesCS3B01G529700.1	2.166814	-1.17498	4.370941
		TraesCS3D01G478000.1	2.297093	-0.34909	3.1826331
		TraesCS3A01G483000.1	2.003146	-0.80249	3.0583785
	*PR5*	TraesCS5A01G017900.1	2.049162	-0.43059	3.7327842
		TraesCS7B01G417700.1	2.559131	-0.37413	2.9550553
		TraesCS5B01G015500.1	3.277238	-0.59929	3.506323
	*PAL*	TraesCS2A01G196700.1	3.974169	0.98228	1.2050875
		TraesCS2B01G224300.1	-	0.493649	1.6846517
		TraesCS6A01G222700.1	-	-	2.4408372
		TraesCS1B01G122800.1	-	0.85019	1.8428717
		TraesCS2D01G204400.1	2.72481	1.084181	2.4505412
		TraesCS2B01G224000.1	3.336739	1.181808	2.8693202
JA	*LOX*	TraesCS4B01G037700.3	-0.46994	0.731112	2.2560122
		TraesCS7D01G244800.1	2.047667	0.277221	2.7179057
		TraesCS2B01G333600.1	0.884414	0.766508	1.1420066
		TraesCS7B01G145200.1	1.751777	0.36088	3.5528851
	*FAD*	TraesCS4A01G109300.1	1.262392	0.733322	2.1290001
		TraesCS2D01G279500.2	0.236077	0.349299	1.3187003
		TraesCS6B01G309400.2	-0.29365	-0.40462	-0.959727
		TraesCS6A01G280000.1	-0.35755	-0.28241	-0.877556
		TraesCS5A01G123600.2	0.46541	0.868997	-0.729088
	*AOC*	TraesCS6D01G314300.1	-	-0.37448	1.0733633
		TraesCS6B01G365200.1	-	-0.2724	1.1269558
		TraesCS6A01G334800.1	-	-0.15234	0.492184
	*JAR1*	TraesCS3A01G145300.1	-0.54738	-0.30317	-
	*PR3*	TraesCS7D01G534700.1	-0.75919	-0.8067	1.7303438
		TraesCS7A01G548100.1	-0.83319	-1.49857	0.5604188
		TraesCS5A01G500200.1	0.166814	-0.91361	0.1453813
	*PR10*	TraesCS5D01G102700.1	-1.83319	-0.95543	1.39692
		TraesCS5A01G090600.1	2.350873	-0.84118	0.7095308
		TraesCS5B01G096300.1	1.324355	-1.47549	1.6880252
	*PDF1.2*	TraesCS1D01G052900.1	0.166814	0.086389	0.1453813

“-” indicates the data wasn’t reliable

JA is widely considered to be antagonistic to SA. Here we found that JA-related genes exhibited a different expression pattern compared with genes involved in SA signaling. Some genes involved in JA synthesis, such as *lipoxygenase* (*LOX*), *allene oxide cyclase* (*AOC*), and *fatty acid desaturase* (*FAD*), were either not up-regulated or down-regulated at all the three time points. However, two *LOX* genes were up-regulated at 0 h, and all four *LOX* genes were up-regulated at 48 hpi. While two *FAD* genes were up-regulated at different time points, the other three were down-regulated. Two *AOC* genes were up-regulated at 48 hpi, but the expression of one *AOC* did not change between compatible and incompatible interactions. The JA-responsive genes *PR3* and *PR10* exhibited variable expression patterns. The transcript levels of *JAR1* and *PDF1.2*, which are thought to be JA-responsive, were not obviously changed (Ruan et al. 2019). The transcript levels of these genes may elucidate the fine-tuned defense network associated with *Yr10*.

### Transcript levels of genes involved in ROS-mediated defense in the response to *Pst* infection

In plants, ROS play a crucial role in not only signaling but also direct defense against pathogens (Mittler et al. 2022; Waszczak et al. 2018). Respiratory burst oxidase homologs (RBOHs) are recognized as the main ROS-producing protein family. Some RBOHs have been found to play a role in the wheat-rust interaction, such as *TaNOX10*(Wang et al. 2022a). Here, we studied the transcript levels of ROS-related genes (Table 2) and found that all three RBOH-type genes, including *TaNOX10*, were significantly up-regulated in the incompatible interaction at 48 hpi. We also found that ROS-scavenging genes such as *alternative oxidases* (*AOX*), *catalase* (*CAT*), *peroxidase* (*POD*), and *superoxide dismutase* (*SOD*), exhibited variable expression patterns. The *AOX* genes were up-regulated at 48 hpi. The majority of *SOD* and *CAT* genes exhibited negligible expression differences between the compatible and incompatible interactions, with the exception of one *SOD* gene and one *CAT* gene which were up-regulated at 48 hpi. The *POD* genes were all significantly up-regulated at 0 hpi and 48 hpi. Notably, some ROS-scavenging genes were down-regulated at 0 hpi.

**Table 2:** Transcript levels of genes in wheat related to ROS

Category	Gene Description	Gene ID	Log_2_ Fold Change		
			0 h	18 h	48 h
ROS	*AOX*	TraesCS2A01G438200.1	-0.9542	-	0.2833548
		TraesCS2A01G438300.1	-	-	1.51
		TraesCS2A01G439100.1	-	-	2.09
		TraesCS2A01G439400.1	-	-0.05286	1.71
	*POD*	TraesCS2B01G125200.1	4.305838	-0.54426	1.7460374
		TraesCS2A01G107500.1	4.669314	-0.5921	1.8433122
		TraesCS2A01G107700.1	6.974169	-0.62483	1.2387778
		TraesCS2B01G124800.1	5.625517	-0.2194	2.238565
		TraesCS2D01G107800.1	5.163665	-0.51166	2.8401733
	*SOD*	TraesCS2D01G123300.1	0.331757	-	0.7797627
		TraesCS2A01G121200.2	0.442514	-	1.0228863
	*CAT*	TraesCS5A01G498000.1	-0.1456	0.093315	-0.623643
		TraesCS6B01G330700.1	-	-0.54423	-0.205624
		TraesCS6A01G041700.1	-1.10868	-	-0.727666
		TraesCS6D01G048300.3	0.415447	-0.15469	1.2283801
	*RBOH*	TraesCS5A01G301700.1	-	-	3.8503436
		TraesCS5B01G299000.1	0.529384	0.242893	3.1767192
		TraesCS5D01G306400.1	-	0.710144	2.705077

“-” indicates the data wasn’t reliable

### Validation of RNA-seq data with qRT-PCR analysis

**Fig. 4:**
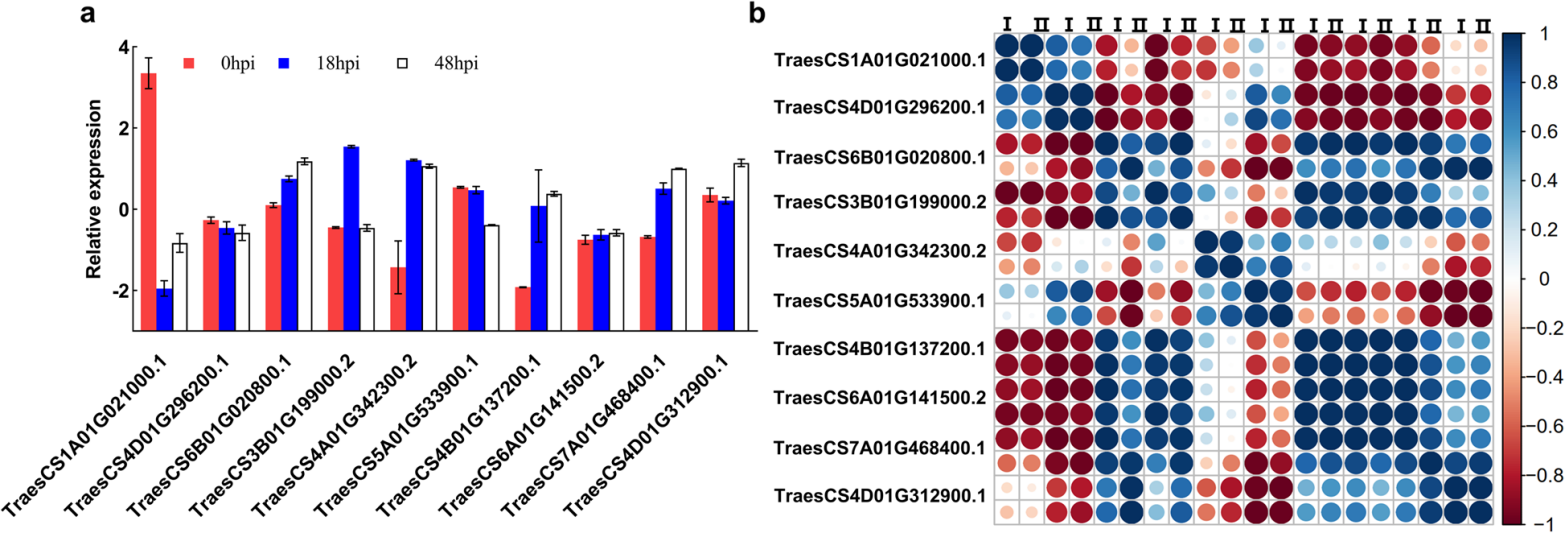
Validation of the RNA sequencing data. **a** Log_2_-transformed relative expression of 10 DEGs by RT-qPCR in AvS vs AvS + *Yr10* at three different time points. **b** Correlation analysis of DEGs between RNA-seq and qRT-PCR data. (I) stands for its Log_2_-transformed gene expression values in qRT-PCR, (II) stands for its Log_2_-gene expression of Illumina, and the colors in the squares are the Pearson correlation coefficients which were calculated by R Studio. Accession number are as below: *TaTI* (TraesCS1A01G021000), *TaCYN* (TraesCS4D01G296200), *TaNOS1* (TraesCS6B01G020800), *TaTPMT* (TraesCS4A01G342300), *TaWRKY* (TraesCS3B01G199000), *TaBHLH* (TraesCS5A01G533900), *TaFER* (TraesCS4B01G137200), *TaHPPD* (TraesCS6A01G141500), *TaGST* (TraesCS7A01G468400), *TaHR* (TraesCS4D01G312900)

To evaluate the reliability of the RNA-seq and DEG analysis, ten candidate genes were selected according to their transcript levels, functions and previous researches, the mRNA levels of candidate genes were analyzed by qRT-PCR (Fig. 4a). The primer sequences are listed in Table S2. The correlation between differential gene expression levels determined by RNA-seq and qRT-PCR was analyzed after log_2_ transformation. The Pearson correlation coefficients for all 10 genes were greater than 0.8, with 7 exhibiting Pearson correlation coefficients greater than 0.95. Overall, the expression trends of the 10 DEGs analyzed by qRT-PCR were consistent with the RNA-seq results, confirming the reliability of the transcriptomic sequencing analysis (Fig. 4b).

### Identification of six candidate genes involved in the response to *Pst* infection

**Fig. 5:**
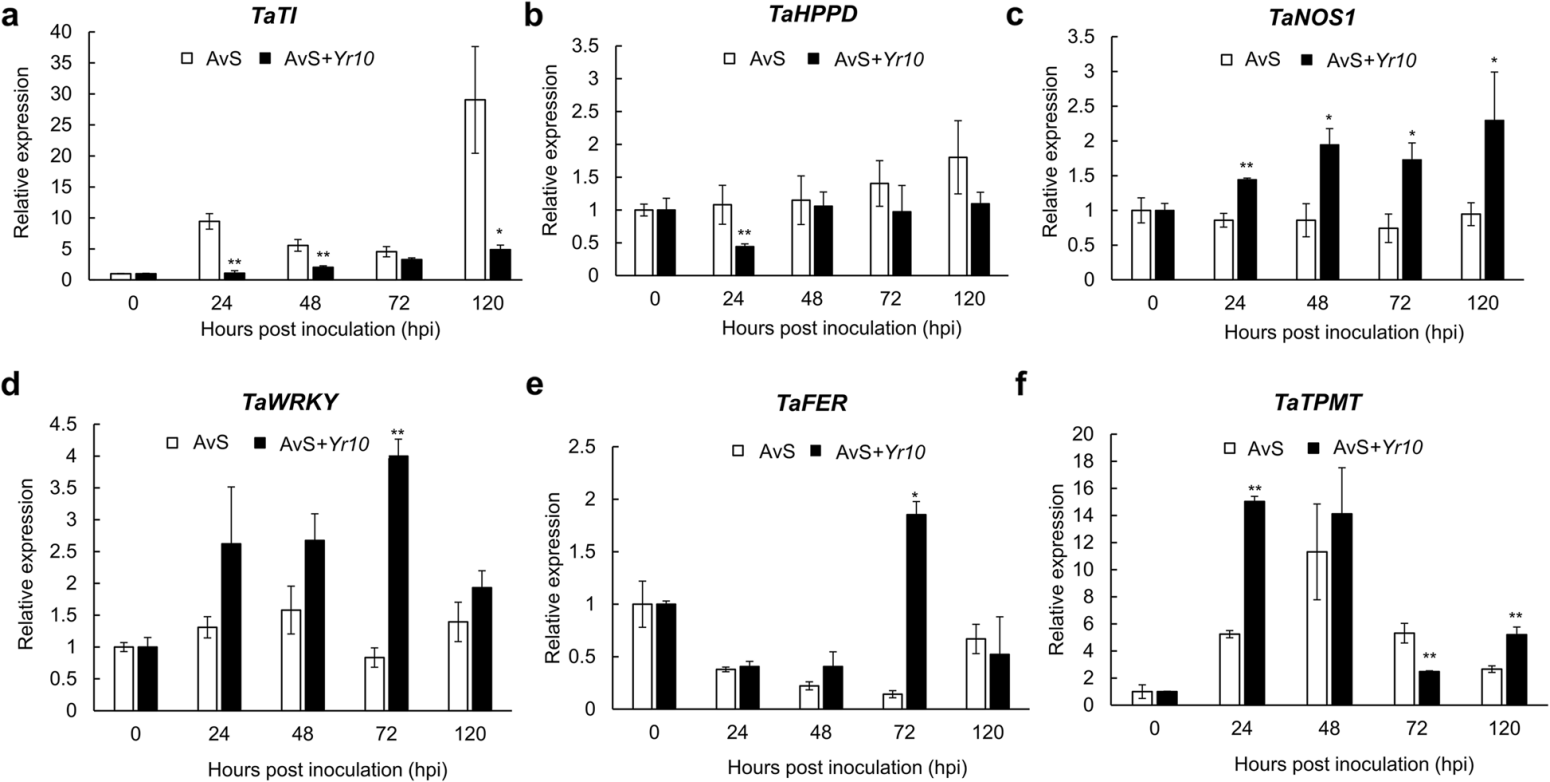
Transcript levels of six candidate genes in AvS (compatible interaction) and AvS + *Yr10* (incompatible interaction) leaves after inoculation with CYR32. Leaf tissues were sampled at 0, 24, 48, 72, and 120 h post-inoculation (hpi). Relative gene expression was calculated by the comparative 2^−ΔΔCt^ method and was relative to the mock at each corresponding time point using gene-specific oligonucleotide primers. Transcript abundance was normalized to the reference gene *TaActin*. The mean expression values were calculated from three replications. Error bars represent standard deviation. (*) and (**) indicates a significant difference between compatible interaction and incompatible interaction with a *p*-value < 0.05 and 0.01, respectively. Differences were assessed using Student’s *t*-tests

As mentioned above, we further identified six candidate DEGs which we deduced they may play a role in the wheat-rust interaction based on their functions. The qRT-PCR analysis showed that the expression levels of two candidate genes, *Trypsin inhibitor* (*TaTI*) and *4-hydroxyphenylpyruvate dioxygenase* (*TaHPPD*), were higher in the compatible interaction than in the incompatible interaction during the *Pst* infection early stage (Fig. 5a, b). At 24 hpi, *TaTI* expression was nearly 8.74-fold and *TaHPPD* expression was nearly 2.43-fold than those in the incompatible interactions, respectively.

In addition, the expression levels of four candidate genes were significantly higher in the incompatible interactions than in the compatible interactions at certain time points, including *nitric oxide synthase 1* (*TaNOS1*), *WRKY transcription factor* (*TaWRKY*), *thiopurine S-methyltransferase family protein* (*TaTPMT*), and *ferritin* (*TaFER*) (Fig. 5c, d, e, f). These results indicate that these candidate genes may play a role in the process of disease resistance. Notably, *TaNOS1*, whose expression level was significantly increased in the incompatible interactions at four different time points, may be essential for wheat disease resistance.

### Functional analysis of the six candidate genes

**Fig. 6:**
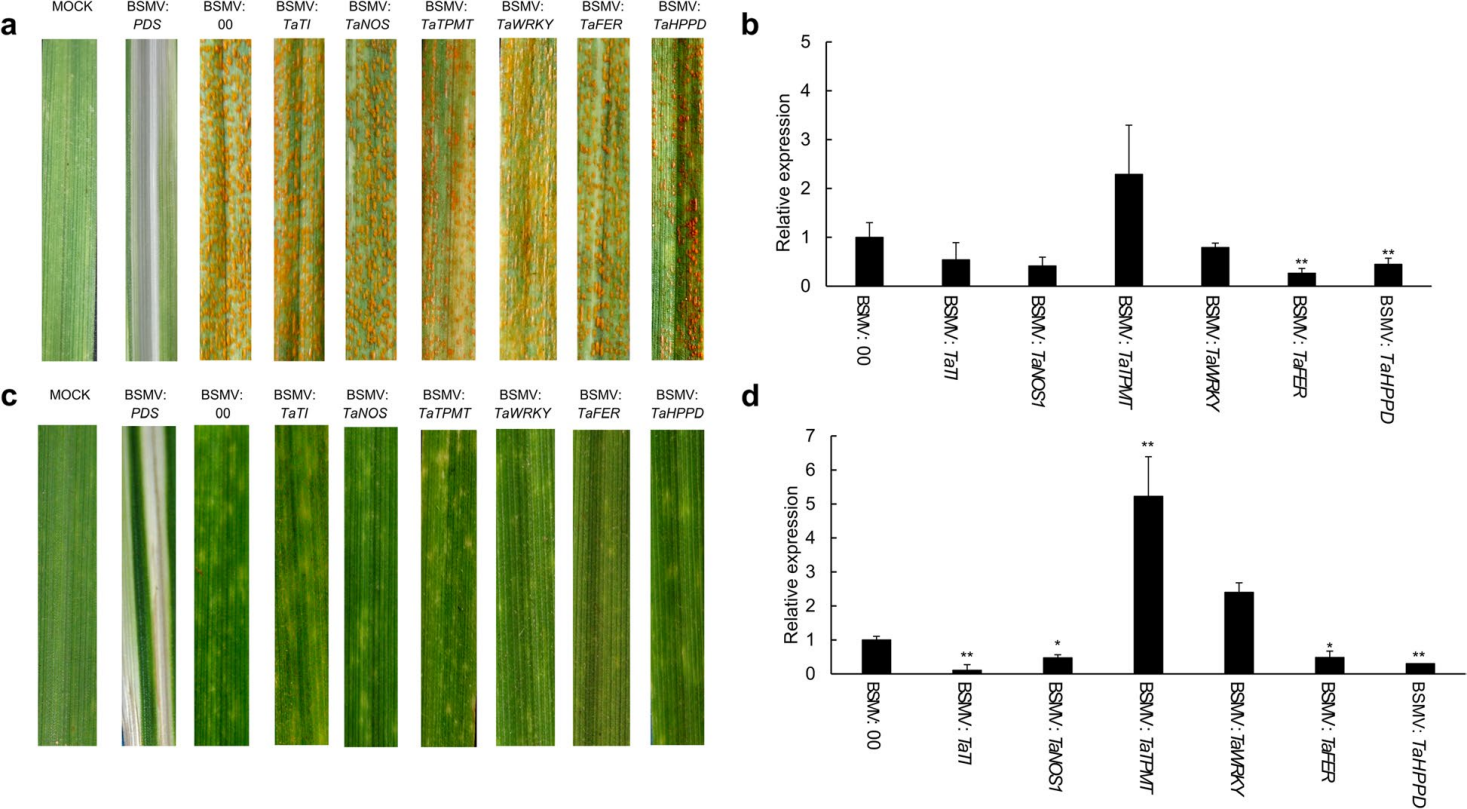
Functional analysis through BSMV-VIGS of six candidate genes in response to *Pst* in compatible and incompatible interactions. The phenotypes were observed on the control (BSMV:00) and candidate gene-silenced AvS (**a**) and AvS + *Yr10* (**c**) wheat leaves at 14 days post inoculation (dpi) with CYR32. Mock wheat leaves were treated with FES buffer (viral inoculation buffer). The photobleaching symptoms on the wheat leaves were photographed at 9 dpi with BSMV: *PDS*. Relative expression of the candidate genes on AvS (**b**) and AvS + *Yr10* (**d**) wheat leaves during the interaction between gene-silenced plants and CYR32. The qRT-PCR values were normalized to reference gene *TaActin* and were presented as fold changes relative to those in plants inoculated with BSMV: 00 at time 0hpi. Data are means ± SD (*n* = 3). Asterisks indicate significant differences between gene-silenced plants and BSMV: 00 according to Student’s *t*-test (*p-value < 0.05, **p-value < 0.01)

We used the barley stripe mosaic virus-induced gene silencing (BSMV-VIGS) assay to knock down the candidate genes in order to investigate the role they play during the process of wheat-pathogen interaction. The six genes, *TaTI, TaHPPD, TaNOS1, TaWRKY, TaTPMT,* and *TaFER*, were silenced in both AvS and AvS + *Yr10* wheat lines. Plants inoculated with a buffer were used as blank controls, including BSMV:00 as a negative control and BSMV: *PDS* as a positive control. The plants inoculated with BSMV: *PDS* exhibited photobleaching at 9 dpi, indicating that the gene silencing induced by BSMV was successful (Fig. 6a). Next, the *Pst* pathotype CYR32 was inoculated on these wheat leaves. Total RNA was extracted at 0 hpi with CYR32 and qRT-PCR was performed to determine the gene silencing efficiency. The results showed the successful silencing of *TaNOS1, TaHPPD,* and *TaFER* in the compatible interactions, and *TaTI, TaNOS1, TaHPPD,* and *TaFER* in the incompatible interactions. Only *TaWRKY* and *TaTPMT* were not silenced in both interactions (Fig. 6b, d).

The phenotype was assessed at 14 dpi with CYR32. In the incompatible interactions, AvS + *Yr10* exhibited high resistance to CYR32 with conspicuous HR symptoms appearing on the BSMV:00 control leaves (Fig. 6c). However, there was no significant difference in plants when other candidate genes were silenced compared with the control. In the compatible interactions, AvS was susceptible to CYR32, and there were numerous urediniospores on the BSMV:00 control leaves (Fig. 6a). Most candidate-gene-silenced plants displayed a similar phenotype to the BSMV:00 control, with the exception that BSMV:*TaHPPD* exhibited a less susceptible phenotype, with fewer urediniospores in contrast to the control. These results indicate that *TaHPPD* may play a negative regulatory role in wheat resistance to *Pst* (Fig. 6a). Taken together, these results suggest that silencing *TaHPPD* diminishes the susceptibility of wheat to stripe rust.

### *TaHPPD-*silenced wheat exhibits reduced susceptibility to stripe rust

**Fig. 7:**
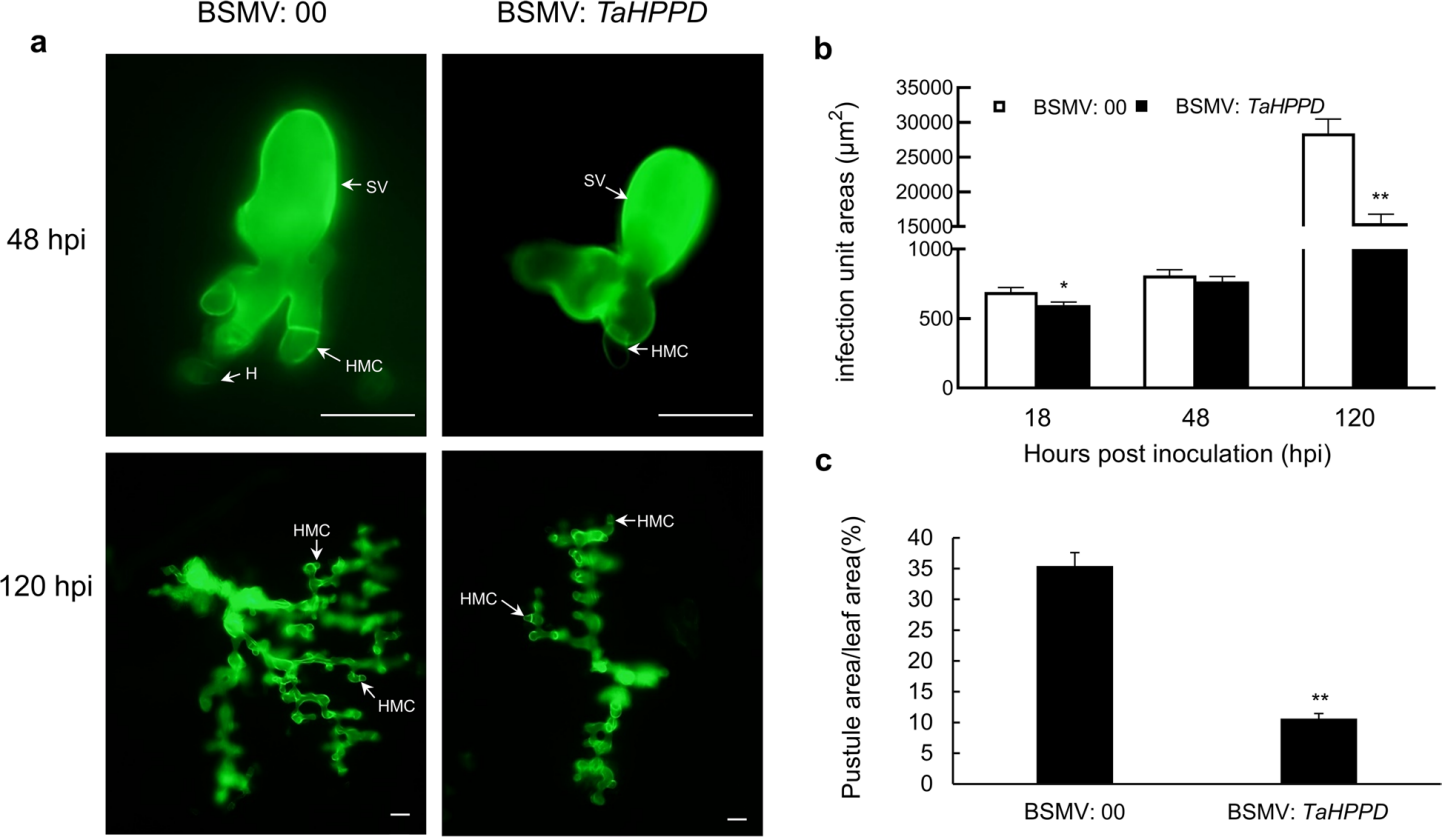
Silencing *TaHPPD* slowed the hyphal growth of *Pst* race CYR32 in the compatible interaction. **a** *Pst* race CYR32 development at 48 h and 120 h post-infection was detected by staining plants with WGA on *TaHPPD* silenced and BSMV: 00 inoculated plants in the compatible interaction (SV, substomatal vesicle; H, Haustoria). Bars = 20 μm. **b** Infection areas were measured microscopically after being stained with WGA in *TaHPPD*-silenced AvS wheat leaves after inoculation with *Pst*. **c** The percentage of the pustule area of the leaf area was measured using Imagine J software. The values are the means ± SD of three biological replicates. Differences were evaluated with Student’s *t*-tests. (*) and (**) show a significant difference with a *p-*value < 0.05 and 0.01, respectively

To verify the impact of silencing *TaHPPD* on *Pst* infection, wheat germ agglutinin (WGA) was used to fluorescently label the infection structures of *Pst* race CYR32 in the leaves of *TaHPPD*-silenced plants in the compatible interaction. The infection was measured histologically using fluorescence microscopy, as shown in Fig. 7a. The results showed that the infected areas of the *TaHPPD*-silenced plants were significantly smaller than the control BSMV:00-inoculated plants at 18 hpi and 120 hpi (Fig. 7b). Meanwhile, the *TaHPPD*-silenced plants displayed significantly (∗ ∗ *P* < 0.01) fewer pustules on their leaves than BSMV:00-inoculated plants (Fig. 7c).

### *TaHPPD* is involved in ROS removal and inhibits the HR in wheat

**Fig. 8:**
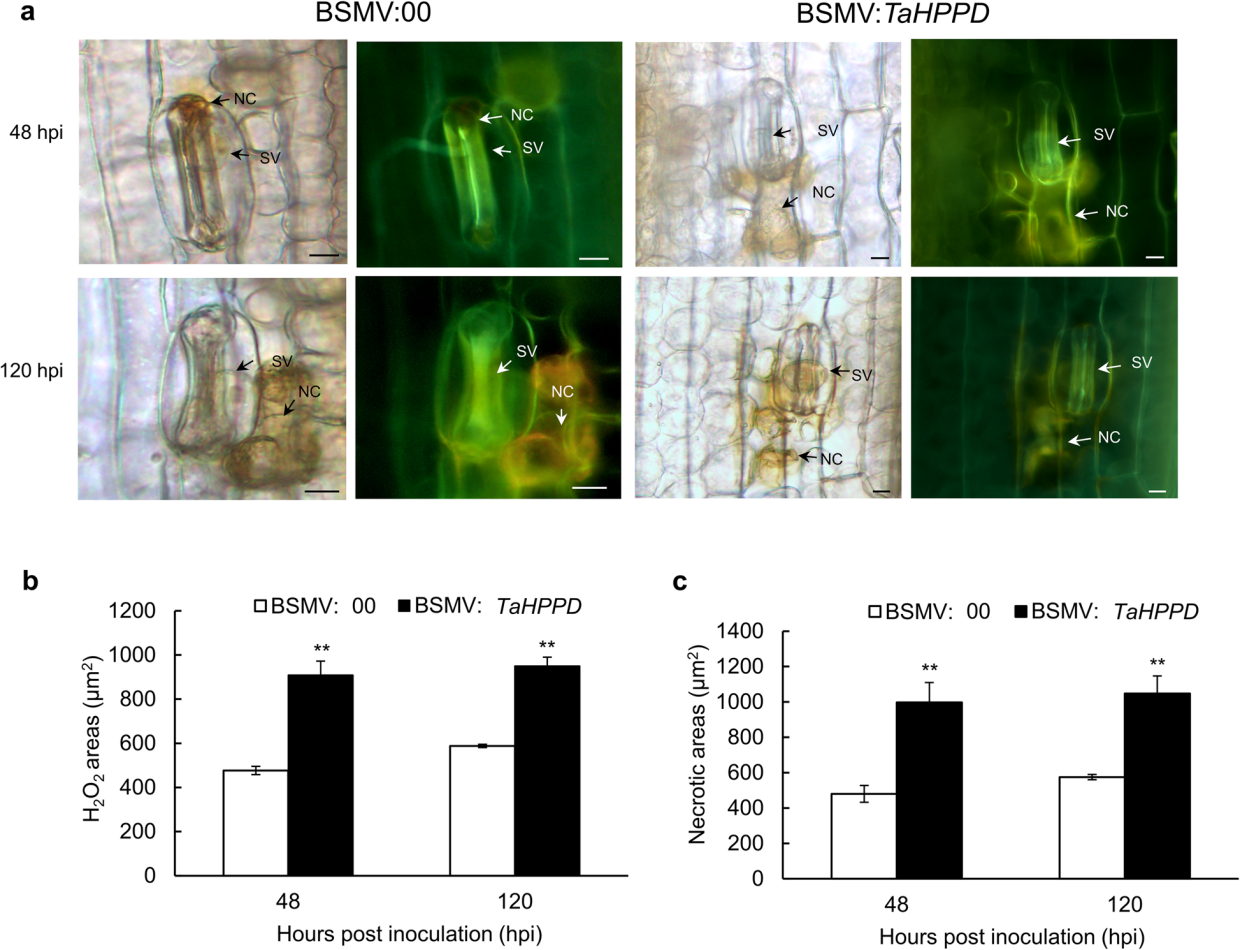
ROS accumulation and hypersensitive response in the *TaHPPD-silenced* leaves and BSMV: 00 leaves after inoculation with *Pst* race CYR32 in the compatible interaction. **a** *TaHPPD*-silenced leaves and BSMV: 00 leaves were stained with DAB and reactive oxygen species areas were measured under a fluorescence microscope at 48hpi and 120 hpi. **b** At 48hpi and 120 hpi, cell death areas were measured in the *TaHPPD*-silenced and BSMV: 00 plants. **c** The reactive oxygen species and cell necrosis triggered by *Pst* were observed under a fluorescent microscope. Images were captured under an epifluorescence microscope at 48hpi and 120 hpi, respectively. BSMV: *TaHPPD* is the experimental group and BSMV: 00 is the control group (NC, necrotic cell; SV, substomatal vesicle), bars = 10 μm. The error bars correspond to the standard deviation among three bio-replicates. Student's *t*-test was used for statistical analysis. (*) and (**) indicate significant differences between the experimental group and the control group, *p-*value < 0.05 and 0.01, respectively

Previous studies have shown that 4-hydroxyphenylpyruvate dioxygenase (HPPD) is an enzyme involved in photosynthesis, and its byproduct homogentisic acid (HGA) is an essential precursor for the biosynthesis of tocopherols. Tocopherols have been found to have an important role in scavenging ROS (Fryer 1992; Ndikuryayo et al. 2017). Therefore, to investigate whether the disease-resistant phenotype of the *TaHPPD-*silenced plants is associated with the elimination of ROS and the inhibition of HR, we examined the accumulation of ROS and the necrotic area in *TaHPPD*-silenced plants and BSMV:00-inoculated plants in the compatible interaction (Fig. 8a).

At 48 hpi and 120 hpi with CYR32, the accumulation of ROS and the necrotic area in BSMV:*TaHPPD* leaves each increased significantly (∗ ∗ *P* < 0.01), compared with BSMV:00 leaves in the compatible interaction (Fig. 8b, c). These results suggest that, due to the possible ROS scavenging ability of *TaHPPD*, the level of hypersensitive cell death and ROS accumulation increased, enhancing the resistance of these plants to *Pst* infection.

### The effect of *TaHPPD* down-regulation on SA, JA, and *PR* genes

**Fig. 9:**
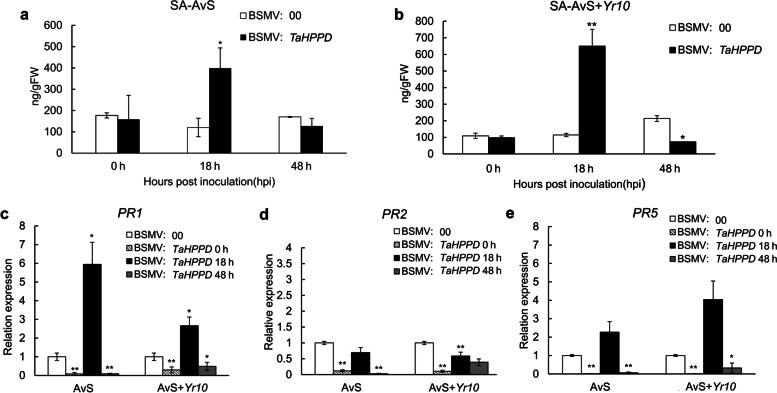
Changes of endogenous SA level and three *PR* genes expression of *TaHPPD*-silenced AvS and AvS + *Yr10* wheat after *Pst* infection. SA concentration in *TaHPPD*-silenced AvS (**a**) and AvS + *Yr10* (**b**) wheat leaves at 0, 18, 48hpi with three biological replicates. *TaPR1* gene expression (**c**), *TaPR2* gene expression (**d**), *TaPR5* gene expression (**e**) in *TaHPPD*-silenced AvS and AvS + *Yr10* wheat leaves at 0, 18, 48 hpi with three biological replicates. The error bars represent the standard deviation among three bio-replicates. Student's *t*-test was used for statistical analysis. (*) and (**) indicated significant differences between certain time point and control, *p-*value < 0.05 and 0.01, respectively

To verify whether the *TaHPPD*-silenced plants’ resistance was associated with SA or JA, we measured the levels of SA/JA and five *PR* genes in *TaHPPD*-silenced AvS and AvS + *Yr10* wheat after *Pst* infection. Primers are listed in Table S2. *TaHPPD-*silenced AvS and AvS + *Yr10* exhibited increased endogenous SA at 18 hpi compared with the control (Fig. 9a, b). At 0 hpi and 48 hpi, *PR1* and *PR5* exhibited significantly (∗ ∗ *P* < 0.01) lower expression levels in *TaHPPD*-silenced AvS and AvS + *Yr10* wheat than in the control. At 18 hpi, the relative expression of *PR1* was significantly higher in *TaHPPD*-silenced AvS and AvS + *Yr10* wheat than in the control (Fig. 9c and e). In addition, the alterations in the expression of *PR1* and *PR5* were in agreement with the changes in SA levels in *TaHPPD*-silenced AvS and AvS + *Yr10* wheat. However, *PR2* exhibited a distinct pattern of expression. Unlike *PR1* and *PR5*, *PR2* was down-regulated at the three time points both in AvS and AvS + *Yr10* compared with the control, which were clearly inconsistent with the level of SA (Fig. 9d).

**Fig. 10:**
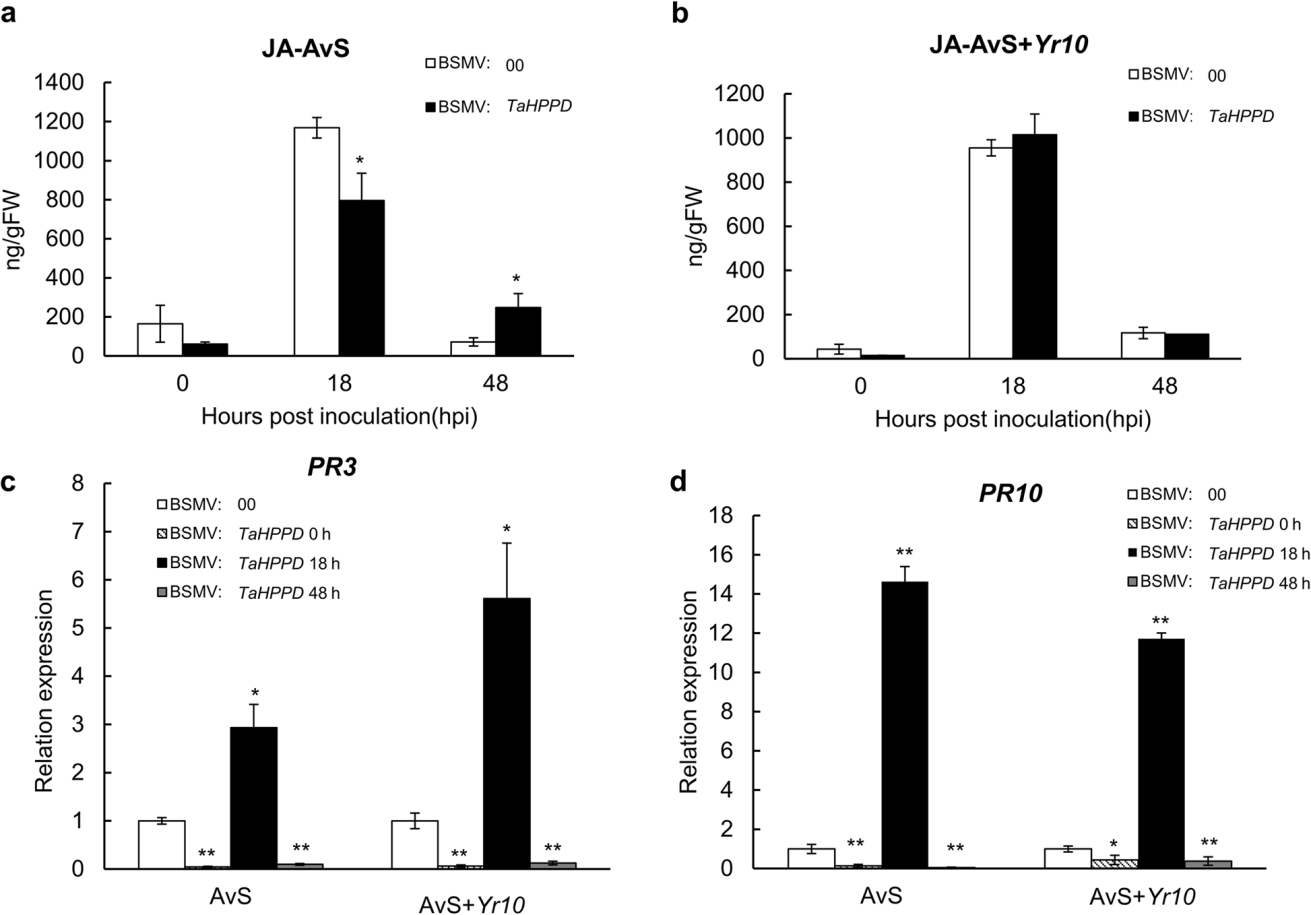
Changes of endogenous JA level and two *PR* genes expression of *TaHPPD*-silenced AvS and AvS + *Yr10* wheat after *Pst* infection. JA concentration in *TaHPPD*-silenced AvS (**a**) and AvS + *Yr10* (**b**) wheat leaves at 0, 18, 48 hpi with three biological replicates. *TaPR3* gene expression (**c**) and *TaPR10* gene expression (**d**) in *TaHPPD*-silenced AvS and AvS + *Yr10* wheat leaves at 0, 18, 48 hpi with three biological replicates. The error bars represent the standard deviation among three bio-replicates. Student's *t*-test was used for statistical analysis. (*) and (**) indicated significant differences between certain time point and control, *p* < 0.05 and 0.01, respectively

In *TaHPPD*-silenced AvS wheat, the JA concentration was significantly lower than in the control at 18 hpi and significantly higher at 48 hpi (Fig. 10a). However, JA concentration exhibited no significant difference at 18 hpi and 48 hpi compared with control in AvS + *Yr10* (Fig. 10b). In the compatible interaction, JA-responsive *PR3* and *PR10* expression were significantly higher at 18 hpi and lower at 48 hpi compared to the control in AvS and AvS + *Yr10* wheat, inconsistent with the JA concentration (Fig. 10c and d). Therefore, due to the SA level increasing significantly in *TaHPPD*-silenced wheat, while JA was not up-regulated, we speculate that SA is involved in the wheat signal transduction network mediated by* TaHPPD.*

## Discussion

### Defense signaling pathways induced in wheat challenged with *Pst*

Plants utilize a complex network of signaling pathways to defend against pathogens, and the cross-talk between the SA and JA signaling pathways is a critical component of this defense mechanism. This crosstalk is beneficial for fine-tuning the defense induction of plants against different plant pathogens. SA plays an important role in the process of plant defense against pathogens, not only in the local defense but also in systemic acquired resistance (SAR) (Spoel et al. 2003). JA is involved in defense against wounds, pests, and microbial pathogens (Kunkel and Brooks 2002). SA and JA accumulate during pathogen infection, leading to the activation of different defense-related genes.

One of the SA biosynthesis pathways that has been studied involves *PAL* (Verberne et al. 1999). We found that all six *PAL* genes were significantly up-regulated at 48 hpi with stripe rust in the incompatible interaction. *NPR1* (also known as *NIM1*) plays a crucial role in the SA-mediated activation of *PR* genes (Cao et al. 1994; Wang et al. 2020). *NPR1* gene expression was upregulated in the *Yr10*-mediated resistance pathway at three different time points. The fold changes of *PR1*, *PR2*, and *PR5* were very high at 48 hpi. These three *PR* genes are commonly used as reporter genes for SA-dependent defense in wheat (Zhang et al. 2018). In addition, endogenous SA levels increased in AvS + *Yr10* after *Pst* inoculation (Zhu et al. 2021). These results suggest that a strong defense pathway was activated by stripe rust at the local and systemic levels, accompanied by the increased endogenous levels of SA in AvS + *Yr10* wheat.

However, the genes involved in the JA signaling pathway exhibited variable expression levels. Some *FAD* genes had moderately increased expression levels upon pathogen infection but others were downregulated during the process, which suggests that the expression of *FAD* genes may be fine-tuned during the defense response. *JAR1*, which is required to activate JA, showed no significant change at 48 hpi compared with the compatible interaction. *PDF1.2*, which is commonly used to monitor JA-dependent defense responses, showed a slight increase in gene expression. *PR3* and *PR10* were upregulated by JA in wheat (Desmond et al. 2005). Though *PR3* and *PR10* were induced at 48 hpi with stripe rust in AvS + *Yr10* wheat, the fold change of transcript level induction was not as high as that of the *PR* gene in the SA pathway. The antagonistic effect of pathogen-induced SA on JA signaling was shown (Spoel et al. 2003). These results suggest that although JA-responsive genes were upregulated, increased SA levels or the SA defense pathway may be the major defense pathway activated by stripe rust.

### Effect of ROS and nitric oxide in the wheat response to *Pst* infection

The initial stage of HR involves a burst of oxidative metabolism that generates superoxide anions (O^2−^) and an accumulation of hydrogen peroxide (H_2_O_2_) (Lamb and Dixon 1997). These ROS can attach to their corresponding receptors and activate plasma membrane calcium influx, and the resulting increased amount of intracellular calcium is involved in the mediation of HR (Wu et al. 2020). The intermediates of these ROS stimulate the expression of defense genes and cell death (Lamb and Dixon 1997). The plasma membrane-localized NADPH oxidases (respiratory burst oxidase homologs, RBOHs) are key ROS-producing enzymes during biotic stress (Yuan et al. 2021; Wang et al. 2022a). In the *Yr10*-mediated resistance immunity pathway, the transcript level of RBOH was significantly up-regulated at 48 hpi, suggesting that this enzyme is crucial for ROS generation and enhanced wheat resistance to stripe rust. The transcript level of ROS-scavenging genes was fine-tuned at different time points between compatible and incompatible interactions. This explains the complex mechanism regulating the clearance and synthesis of ROS. H_2_O_2_ accumulation has been observed in wheat stoma cells and mesophyll cells after inoculation in *Yr10*-mediated interaction with stripe rust (Zhu et al. 2021), implying that the host resistance gene (*Yr10*) can regulate disease resistance by affecting the accumulation of ROS.

Nitric oxide (NO) has been shown to participate in the response of plants to biotrophic and hemibiotrophic pathogens, and affects both basic defense and HR (Mur et al. 2005). In the interaction between wheat and stripe rust, NO as an important signaling molecule involved in the regulation of *Pst* urediniospore germination (Yin et al. 2016). Endogenous NO synthesis is dependent on NO synthase activity. We had identified one DEG, *TaNOS1* which participates in NO synthesis and were up-regulated at 48 hpi. The transcript level of *TaNOS1* was found to be significantly increased in the incompatible interactions at four different time points by RT-PCR analyses, indicating that this gene may be necessary for wheat disease resistance. The interaction between the NO and ROS signaling pathways is essential for regulating stress responses, and the balance of NO/ROS levels can influence the resistance of wheat to stripe rust (Yin et al. 2016). Moreover, NO can trigger the production of SA, which is involved in the activation of SA-dependent gene expression (Mur et al. 2013). Collectively, the ROS and NO defense pathways may have complicated interactions in the *Yr10*-mediated signaling pathway.

### Photosynthesis also plays a part in wheat resistance to *Pst*

Photosynthesis provides the materials and energy necessary for various physiological metabolic processes and is closely related to plant defense response (Göhre et al. 2012). Studies have suggested that photosynthesis may be involved in the immune defense response of wheat against fungal pathogens (Lu and Yao 2018; Kangasjärvi et al. 2014). For example, wheat stripe rust effector proteins can reduce photosynthesis and inhibit the production of chloroplast-derived ROS (Xu et al. 2019). When fighting against an invading pathogen, resistant wheat genotypes can proactively modulate photosynthetic changes in order to reduce pathogen infection through the initial suppression of photosynthesis-associated gene expression (Bolton 2009).

Wang et al.(2019) found that by inhibiting photosynthesis, the stripe rust resistance gene *Yr36* can provide broad-spectrum resistance to *Pst* races in mature wheat. To reveal whether *Yr10*, another stripe rust resistance gene, exerts its resistance effect through manipulating photosynthesis, this study annotated all DEGs and analyzed the metabolic pathways enriched in those DEGs. Interestingly, the results showed that photosynthesis-related processes and components, such as antenna proteins and carbon fixation, were enriched at all three time points. Therefore, we speculate that *Yr10* may enhance resistance by regulating photosynthesis in the early stages of stripe rust infection.

### Regulation of iron is as a strategy to defend against pathogens

Among the DEGs identified between AvS.18 h vs. AvS + *Yr10*.18 h, some up-regulated DEGs were found to be enriched in GO terms associated with iron regulation, including intracellular sequestering of iron ion, iron ion transport, iron-sulfur cluster binding, metal ion binding, and iron homeostasis process. Iron-sulfur clusters are an essential component of mitochondria, participating in ATP production, cofactor biosynthesis, and apoptosis, among other processes. The enrichment results showed that wheat plants may protect themselves through the regulation of iron and downstream processes affected by iron. Because excessive iron is toxic for most organisms, iron balance is tightly controlled to maintain a state of iron homeostasis (Lill and Freibert 2020). Perturbation of iron homeostasis has been used as a strategy by both the host and pathogen to defend and attack, respectively (Verbon et al. 2017). Plants can over-accumulate iron around infection sites or deprive pathogens of iron to defend themselves (Liu et al. 2021). One candidate gene, *TaFER*, which encodes a kind of ferritin, was enriched in the GO term iron homeostasis process and was up-regulated at 18 hpi and 48 hpi according to the RNA-Seq data and qRT-PCR results. Thus, we speculate that this gene may be associated with disease resistance in the interaction between wheat and stripe rust. To verify our speculation, we used the BSMV-VIGS knockdown approach to determine whether *TaFER* plays a significant role during wheat-rust interaction. The silencing efficiency of *TaFER* was above 50%, but the phenotypic changes were not apparent. Even so, we speculate that iron regulation may be a strategy used by wheat at during the early stages of pathogen infection. Although plants may take advantage of iron and components or processes regulated by iron to defend themselves, the candidate gene *TaFER* may have little effect on defending wheat against *Pst*, as the phenotype of the silenced wheat line was not reversed.

### *TaHPPD* may negatively regulates the defensive response

Previous studies found that HPPD promotes the conversion of 4-hydroxyphenylpyruvate (HPP) to HGA (Ren et al. 2011). In plants, HGA plays a significant role as a precursor in the tocopherol biosynthetic pathway (Ren et al. 2011). Tocopherols are crucial antioxidants in plants, and they help to protect the integrity of membrane lipids by scavenging ROS (Matringe et al. 2008). During pathogen attack, ROS and SA can collaborate synergistically to mediate the HR, which is a defense mechanism against pathogen invasion. Here, we found that both ROS accumulation and necrotic area were significantly higher in the BSMV:*TaHPPD* AvS wheat at 48 hpi and 120 hpi, compared to the control. Additionally, compared with the BSMV:00 plants, the spore production was delayed and the number of spores was significantly reduced in the *TaHPPD-*silenced AvS cultivar. SA concentration was also significantly increased at 18hpi in both *TaHPPD-*silenced AvS and AvS + *Yr10* wheat. We speculate that *TaHPPD* silencing may indirectly affect the ROS and SA signaling pathway to enhance plant resistance to *Pst*. Interesting, Wang et al. (2022a) discovered that *TaWRKY19* is a wheat susceptibility factor whose induction minimizes ROS generation, thus compromising plant immunity against the pathogen. And after, another susceptible receptor-like cytoplasmic kinase gene, *TaPsIPK1*, was found to impair wheat immunity and knocking out this gene conferred broad spectrum resistance to *Pst* without effecting wheat agriculture traits (Wang et al. 2022b). Susceptible genes have anticipating application prospect as a novel strategy in breeding which broad our breeding strategy instead of just focusing on resistance genes. Together, we considered that *TaHPPD* was also another susceptible gene in wheat as knocking down *TaHPPD* lead to SA and ROS increasing, further conferred resistance to *Pst*.

## Materials and methods

### Plant materials and* Pst* inoculation

Wheat cultivars AvS and its near-isogenic line containing the resistance gene *Yr10* (AvS + *Yr10*) were used in this study. Wheat seedlings planted in a growth chamber at 15 °C under a 16 h light /8 h dark photoperiod. The inoculation of stripe rust adopted the spray inoculation method. A video protocol detailing the process can be accessed at https://vimeo.com/48605764. The fresh uredospore strain was diluted in Isohexadecane (IHD) to make a spore suspension (Zhu et al. 2021). Then the spore suspension is sprayed on the seedlings at the 2-leaf stage with an air pump. After inoculation, it was maintained in a 100% humidity chamber for 24 h and then cultured in the growth chamber under the same condition as above.

### cDNA library construction and sequencing

Total RNA was extracted using the Rapid Universal Plant RNA Extraction Kit (Huayueyang Biotechnology, Beijing, China) and then analyzed with 2100 Bioanalyzer (Agilent, USA). The qualified RNA was digested by DNase I (Takara, Japan) at 37℃ for 30 min to remove the residual DNA. The digested RNA was purified for mRNA using Dynabeads® Oligo (dT)25 (Life, USA). The cDNA library was constructed using NEBNext® UltraTM RNA Library Prep Kit for Illumina (NEB, US). TruSeq PE Cluster Kit (Illumina, USA) was used to carry out cluster generation in cBot, and then two-way sequencing was performed in Illumina HiseqTM4000 (Illumina, USA). The raw reads were filtered by Trimmomatic v0.32 (Bolger et al. 2014) and evaluated through Fast-QC (Andrews et al. 2010) software to get clean reads for further analysis.

### Gene expression level and differential gene analysis

The generated clean reads were aligned to the wheat reference genome of “Chinese Spring” (IWGSC RefSeq v1.0) (IWGSC, 2018) using Bowtie2 version 2.2.5 (Langmead and Salzberg 2012). The level of gene expression was measured by the number of reads that were aligned to the reference sequence. To eliminate the effects of different gene lengths and sequencing depths on read count, the Reads Per Kilobase Million (RPKM) was used to evaluate gene expression levels (Mortazavi et al. 2008). Differential gene analysis was performed using the MARS (MA-plot-based method with Random sampling model) model in the DEG seq V1.20.0 package (Wang et al. 2010). Genes expression differences between the sample were identified as significantly differentially expressed if they meet the following terms at the same time: | log_2_ Fold-Change |> 2, FDR (Q-value) < 0.001, at least one sample RPKM > 20.

### Gene Ontology and KEGG analysis

Through GO enrichment analysis of differential genes, the server calculates the hypergeometric distribution relationship between these differential genes and specific branches in GO classification and annotated them to corresponding GO entries (Young et al. 2010). In this study, DAVID online website was used for Go enrichment analysis (Sherman et al. 2022).

KEGG significance enrichment analysis takes the KEGG pathway as the unit and uses a hypergeometric test to find out the pathways that are significantly enriched in DEGs compared with the background. The *p*-value is checked and corrected by the BH method to obtain the FDR value. Taking FDR-corrected Q-values ≤ 0.05 as the threshold, GO terms and KEGG pathways meeting this condition were identified as significantly enriched.

### Quantitative real-time PCR (qRT-PCR)

Wheat cultivar AvS and AvS + *Yr10* inoculation with *Pst* race CYR32 were sampled at 0, 24, 48, 72, and 120 hpi. Samples were quickly frozen in liquid nitrogen. Frozen samples were ground using a TissueLyser II (QIAGEN, Germany). The Rapid Universal Plant RNA Extraction Kit (Huayueyang Biotechnology, Beijing, China) was used to extract and purify the total RNA. Then, RNA was reverse transcribed into cDNA by using the Revert Aid First Strand cDNA Synthesis Kit (Thermo Fisher Scientific, United States).

To analyze the expression patterns of the candidate genes after inoculation with *Pst* race CYR32, ten pairs of primers were used for quantitative real-time PCR (qRT-PCR). Quantitative primers were designed by Premier 5.0 software and all primers were listed in Supplementary Table S1. To standardize the data, wheat actin gene was used as a reference gene. The qRT-PCR analysis was performed on CFX96 Real-Time System (Bio-Rad, Munich, Germany) according to the manufacturer's guidelines. Each qRT-PCR reaction system was performed in a 20 μL volume including 10 μL of 2 × ChamQ SYBR qPCR Master Mix (Vazyme, Nanjing, China), 1 μL of each primer, 2 μL of template cDNA, and 6 μL of ddH_2_O. Reactions without templates were utilized as negative controls, and each reaction was executed in triplicate. Three independent biological replicates were performed for each time point and treatment. The relative quantitative 2^− ΔΔ CT^ method was used to analyze the experimental result (Livak and Schmittgen 2001).

### BSMV-mediated gene silencing

To silence the six candidate genes in wheat cultivar AvS and AvS + *Yr10,* six conserved regions of about 200 bp were selected as the silencing segment, respectively. Silencing plasmids were constructed by connecting the target segments with the linearized γ-PCR vector. The mMachine T7 in vitro transcription kit (Ambion, Austin, TX, USA) was utilized to generate in vitro transcripts from linearized tripartite BSMV genome. The experiment used Holzberg’s method (Holzberg et al.^2002^). The proportion of each genome at the time of inoculation is α: β: γ / γ- *PDS* / γ-Target gene = 1:1:1, every 3μL of mixed liquid plus 7μL FES buffer, used to rub-inoculate the first and second leaf of the two-leaf stage wheat seedlings. The construct carrying a *PDS* (phytoene desaturase) was utilized as a positive control, and only the BSMV genome was used as a nontarget negative control and labeled as BSMV: 00. Infected plants were cultured in a controlled chamber with a temperature of 25–28 ℃ and a 16/8 h light/dark photoperiod. After 12d of virus inoculation, the fourth leaf was inoculated with *Pst* isolate CYR32. The phenotype of the fourth leaf was observed and photographed 12–15 days after inoculation. The experiment repeated at least three times.

### Histochemical assays

BSMV: *TaHPPD* and control leave samples of 48 and 120 hpi were observed for ROS accumulation by DAB staining and HR response areas by autofluorescence of necrotic cells under an Olympus BX-51 fluorescence microscope (Olympus Corp., Tokyo, Japan). For the DAB staining experiment, the infected wheat leaves were submerged in the 1 mg/mL DAB solution for 8 h under bright light. After immersed in the buffer the leaves were cut up, and decolored with the decolorizing solution (absolute ethyl alcohol: acetic acid, 1:1 v/v).

As previously described method (Wang et al. 2017), wheat germ agglutinin (WGA) coupled with Alexa 488 (Sigma-Aldrich, Shanghai, China) fluorescein was used to stain the infection structure of *Pst* in *TaHPPD*-silencing plant and control. For each treatment, at least 30 infection sites were examined in 5–10 randomly selected leaf segments. Substomatal vesicles were regarded as successful penetration at infection sites. Using the SPSS program (SPSS, Inc. Chicago, United States), standard deviations were calculated and the Tukey's test was executed for statistical analysis.

### SA/JA level analysis with the LC–MS

To analyze the change of endogenous SA/JA concentration in the compatible and incompatible interactions after inoculation with *Pst* race CYR32, fresh leaves were sampled at 0, 18, and 48hpi. The extraction of SA was according to Segarra et al.’s (2006) method and modified as followed. Standards of salicylic acid > 99% (Fluka, Buchs, Switzerland) and ( ±)-jasmonic acid > 97% (Sigma–Aldrich, Steinheim, Germany) were prepared at a concentration of 500 mg l^−1^ in MeOH. Frozen samples were ground by mortar and pestle in the liquid N_2_ and extracted by 750 µL mixture of MeOH: H_2_O: HOAc (90:9: 1, v/v/v). The solutions were centrifuged at 9000 g speed for 1 min, transfered the supernatant into a new centrifugal tube, and repeated three times. Then the supernatant was dried in N_2_, then dissolved in 1000 μL of pure chromatographic grade MeOH. Finally, the solution was filtered for a Millex-HV 0.22 μm filter (Millipore, Bedford, MA, USA).

Three biological replicates were conducted in each assay. SA/JA content was measured using an Agilent 1260 Infinity II LC Campaign (Agilent, USA) machine in the State Key Laboratory of Crop Stress Biology for Arid Areas, NWAFU, China.

### Quantification of pustule density on leaves

Imagine J was used to measure the percentage of the pustule area of the leaf area. The website of Imagine J is (http://rsb.info.nih.gov/ij).

### Supplementary Information

**Additional file 1: Figure S1.**

**Additional file 2: Figure S2.**

**Additional file 3: Figure S3.**

**Additional file 4: Table S1** and **Table S2.** 

## Data Availability

All data generated or analyzed during this study are included in this published article and its supplementary information files.

## Abbreviations

DEGs: Differentially expressed genes
GO: Gene Ontology
SA: Salicylic acid
JA: Jasmonic acid
KEGG: Kyoto Encyclopedia of Genes and Genomes
WGA: Wheat germ agglutinin
SV: Substomatal vesicle
IH: Infection hyphal
NC: Necrotic cell
HMC: Haustorial mother cells
ROS: Reactive oxygen species
PDS: Phytoene desaturase
qRT-PCR: Quantitative reverse transcription-PCR
APR: Adult plant resistance
ETI: Effector-triggered immunity
NBS-LRR: Nucleotide-binding leucine-rich-repeat receptors
HR: Hypersensitive response
scRNA-seq: Single-cell RNA sequencing
IHD: Isohexadecane
hpi: Hours post inoculation
RPKM: Reads per kilobase per million mapped reads
*PAL*: *phenylalanine ammonia-lyase*
*LOX*: *lipoxygenase*
AOC: *allene oxide cyclase*
*FAD*: *fatty acid desaturase*
AOX: *alternative oxidases*
*CAT*: *catalase*
*POD*: *peroxidase*
*SOD*: *superoxide dismutase*
HPPD: 4-Hydroxyphenylpyruvate dioxygenase
BSMV-VIGS: Barley stripe mosaic virus-induced gene silencing
HGA: Homogentisic acid
SAR: Systemic acquired resistance
H_2_O_2_: Hydrogen peroxide
NO: Nitric oxide
