# Supplementary figures and images for: Transcriptomic analysis of wheat reveals possible resistance mechanism mediated by Yr10 to stripe rust
Source: Stress Biol. 2023 Oct 23;3(1):44. doi: 10.1007/s44154-023-00115-z (PMC10593697; doi:10.1007/s44154-023-00115-z)

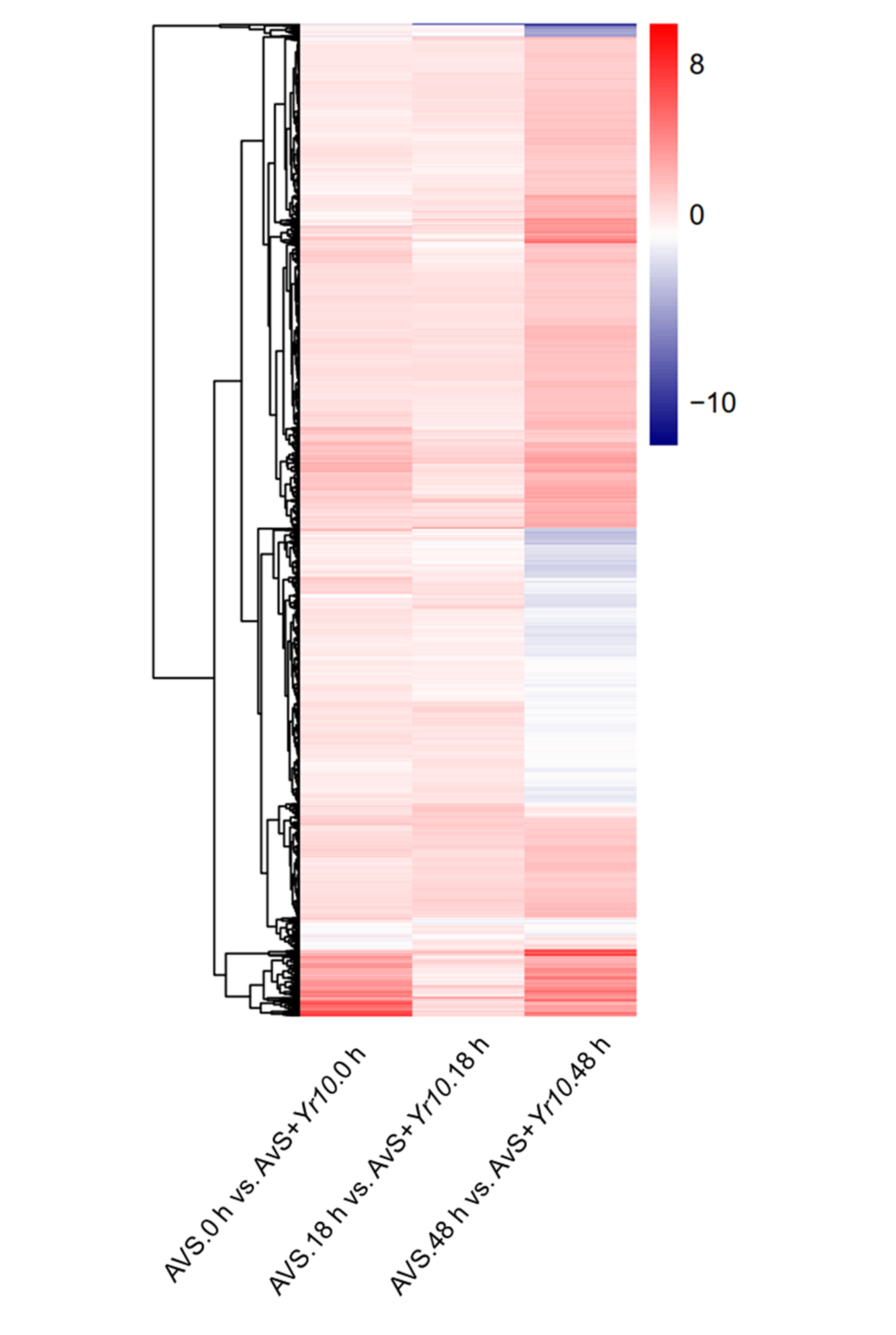

Supplement: Supplementary file 1 — Additional file 1: Figure S1. [file 44154_2023_115_MOESM1_ESM.tif]

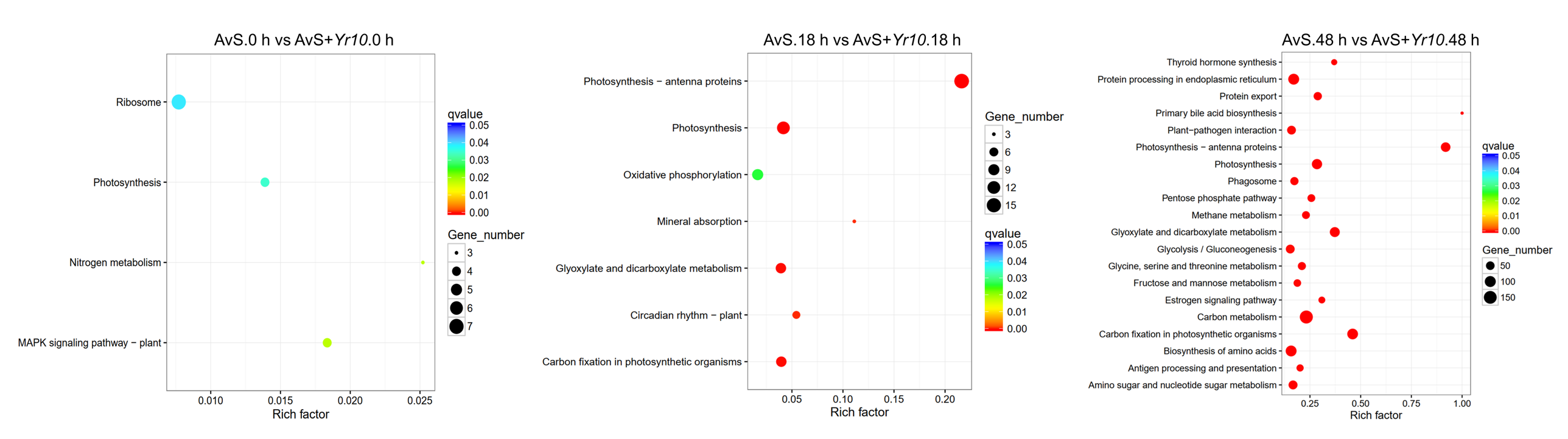

Supplement: Supplementary file 3 — Additional file 3: Figure S3. [file 44154_2023_115_MOESM3_ESM.tif]
