# Supplementary material for: Transcriptomic analysis of wheat reveals possible resistance mechanism mediated by Yr10 to stripe rust
Source: Stress Biol. 2023 Oct 23;3(1):44. doi: 10.1007/s44154-023-00115-z (PMC10593697; doi:10.1007/s44154-023-00115-z)
Supplement: Supplementary file 4 — Additional file 4: Table S1 and Table S2. [file 44154_2023_115_MOESM4_ESM.docx]

Table S1 Results of quality control analysis

| Sample Name | Raw reads | Clean reads | Average length(bp) | Raw data | Clean data | Read 1 Q20 | Mapping to CS ratio | Read 1 GC content |
| --- | --- | --- | --- | --- | --- | --- | --- | --- |
| 1.AvS.0h | 32781101 | 31252232 | 1*150 | 4.92G | 4.69G | 99.05% | 76.08% | 53.60% |
| 2.AvS.0h | 37077715 | 35273242 | 1*150 | 5.56G | 5.29G | 99.04% | 75.62% | 53.32% |
| 3.AvS.0h | 35067083 | 33428535 | 1*150 | 5.26G | 5.01G | 99.12% | 75.78% | 53.43% |
| 4.AvS+*Yr10*.0h | 33788141 | 32209956 | 1*150 | 5.07G | 4.83G | 99.13% | 72.88% | 51.40% |
| 5.AvS+*Yr10*.0h | 32796536 | 31069371 | 1*150 | 4.92G | 4.66G | 99.01% | 72.09% | 51.11% |
| 6.AvS+*Yr10*.0h | 31663772 | 29967103 | 1*150 | 4.75G | 4.50G | 99.01% | 72.20% | 51.12% |
| 7.AvS.18h | 35703137 | 33816540 | 1*150 | 5.36G | 5.07G | 99.03% | 74.12% | 53.67% |
| 8.AvS.18h | 35606657 | 33749239 | 1*150 | 5.34G | 5.06G | 99.02% | 73.82% | 53.52% |
| 9.AvS.18h | 33524609 | 31725361 | 1*150 | 5.03G | 4.76G | 98.92% | 73.59% | 53.51% |
| 10.AvS+*Yr10*.18h | 35748463 | 33857630 | 1*150 | 5.36G | 5.08G | 98.94% | 71.38% | 52.25% |
| 11.AvS+*Yr10*.18h | 30795225 | 29181486 | 1*150 | 4.62G | 4.38G | 98.94% | 71.47% | 52.26% |
| 12.AvS+*Yr10*.18h | 35060693 | 33397858 | 1*150 | 5.26G | 5.01G | 99.04% | 71.97% | 52.54% |
| 13.AvS.48h | 34321790 | 32455046 | 1*150 | 5.15G | 4.87G | 98.89% | 72.48% | 53.94% |
| 14.AvS.48h | 35077372 | 33268870 | 1*150 | 5.26G | 4.99G | 98.96% | 72.39% | 53.94% |
| 15.AvS.48h | 39142297 | 37244787 | 1*150 | 5.87G | 5.59G | 99.09% | 72.80% | 54.03% |
| 16.AvS+*Yr10*.48h | 36084075 | 34386288 | 1*150 | 5.41G | 5.16G | 99.10% | 66.38% | 51.55% |
| 17.AvS+*Yr10*.48h | 37571330 | 35807093 | 1*150 | 5.64G | 5.37G | 99.11% | 66.36% | 51.61% |
| 18.AvS+*Yr10*.48h | 33025647 | 31486731 | 1*150 | 4.95G | 4.72G | 99.10% | 66.59% | 51.61% |

Table S2 Primers used in this study for qRT-PCR

| Name | Primer sequence（5’-3’） |
| --- | --- |
| Ta*TI*-qRT-F | CAGGTGCCAGGATGTGTC |
| Ta*TI*-qRT-R | GCTCGCAGAAGTTGGTGAT |
| Ta*CYN*-qRT-F | GAGCCCGCCATATACAGA |
| Ta*CYN*-qRT-R | GTGAGGCAGATACTTCCCA |
| Ta*NOS1*-qRT-F | GGGCAAGCAGTTCGTTTCG |
| Ta*NOS1*-qRT-R | GTGTCTCTGGGTAGGAGGTCAA |
| Ta*TPMT*-qRT-F | TCATCACCGCCGACTTCT |
| Ta*TPMT*-qRT-R | TTGAGCACCTCCTCGTAGC |
| Ta*WRKY*-qRT-F | ACCGTGCTTCCGTGCTC |
| Ta*WRKY*-qRT-R | GGTCGGGCGACGTTTAG |
| Ta*BHLH-*qRT-F | GCTTGGATTTCTCTTGGGA |
| Ta*BHLH*-qRT-R | CTGGATGATGGACGCCTT |
| Ta*FER*-qRT-F | AAGGGGAAGGAGGTGCT |
| Ta*FER*-qRT-R | TAGGCGAAGAGGGAGTGAT |
| Ta*HPPD*-qRT-F | GGCGTGAGCAACCCAG |
| Ta*HPPD*-qRT-R | CGTCCTCCGTCGTGAACT |
| Ta*GST*-qRT-F | CCCTCAAAGGCGTGGAA |
| Ta*GST*-qRT-R | TCAACCCGATAACAGCGTAA |
| Ta*HR*-qRT-F | CTGGCTCTCTACCTTGCTTTC |
| Ta*HR*-qRT-R | CTCTTGGGGACCGAGTTCTT |
| TaActin-qRT-F | TGACCGTATGAGCAAGGAG |
| TaActin -qRT-R | CCAGACAACTCGCAACTTAG |
| TaTI-vigs-F | GCCTCAGGCGCTGCTCCT |
| Ta*TI*-vigs-R | TCGGCGACACATCCTGGCAC |
| Ta*NOS1*-vigs-F | AGGATGGTGTGGGCTTC |
| Ta*NOS1*-vigs-R | ACCTCGACTGGTTTGGG |
| Ta*TPMT*-vigs-F | CTCCAACCCGGCCATC |
| Ta*TPMT*-vigs-R | GCCACCGCGTCGTATC |
| Ta*WRKY*-vigs-F | GCTGACGTGGCGGAAGA |
| Ta*WRKY*-vigs-R | GCGTGGGCAAGGGTTT |
| Ta*FER*-vigs-F | ATCACTCCCTCTTCGCCTAC |
| Ta*FER*-vigs-R | GTTTCTCATTCACCAGCTTTTC |
| Ta*HPPD*-vigs-F | TCAAGGAATGCCAGGAGC |
| Ta*HPPD*-vigs-R | GAGAAGTTGCCTTTGCCG |
| PR1F | CTGGAGCACGAAGCTGCAG |
| PR1R | CGAGTGCTGGAGCTTGCAGT |
| PR2F | CTCGACATCGGTAACGACCAG |
| PR2R | GCGGCGATGTACTTGATGTTC |
| PR3F | AGAGATAAGCAAGGCCACGTC |
| PR3R | GGTTGCTCACCAGGTCCTTC |
| PR5F | ACAGCTACGCCAAGGACGAC |
| PR5R | CGCGTCCTAATCTAAGGGCAG |
| PR10F | TTAAACCAGCACGAGAAACATCAG |
| PR10R | ATCCTCCCTCGATTATTCTCACG |
